# Sex differences in the association between cumulative social risk and life’s essential 8 cardiovascular health scores in U.S. adults

**DOI:** 10.1016/j.ajpc.2026.101566

**Published:** 2026-03-17

**Authors:** Faith E. Metlock, Ketum Ateh Stanislas, Oluwabunmi Ogungbe, Biruk Teshome, Thomas Hinneh, Ruth-Alma Turkson-Ocran, Cheryl R. Himmelfarb, Garima Sharma, Yvonne Commodore-Mensah

**Affiliations:** a Johns Hopkins School of Nursing, Baltimore, MD, USA; b Johns Hopkins Bloomberg School of Public Health, Baltimore, MD, USA; c Department of Medicine, Research Section, Beth Israel Deaconess Medical Center, Boston, MA USA; d Inova Heart and Vascular Institute, Inova Fairfax Medical Campus, Fairfax, VA, USA

**Keywords:** Life's Essential 8, Cardiovascular health, Social determinants of health, Cumulative social risk, Sex differences, Health equity, Cardiovascular disparities

## Abstract

**Introduction:**

Cardiovascular health (CVH) disparities persist in the U.S., often shaped by social risk factors. However, few studies have examined how individual and cumulative social risks relate to CVH across sex.

**Methods:**

We analyzed cross-sectional data from the 2013–2023 National Health and Nutrition Examination Survey (NHANES). CVH was defined by Life’s Essential 8 (LE8) which includes diet, physical activity, nicotine exposure, sleep, body mass index, blood lipids, blood glucose, and blood pressure. LE8 scores range from 0 to 100 and were categorized as low (<50), moderate (50–79), or high (≥80). Social risk was defined across six domains: low income, low education, minoritized race or ethnicity, single-living status, lack of health insurance, and unemployment, summed and categorized as 0, 1, 2, 3, or ≥4 risks. Weighted multinomial logistic regression models adjusted for age were used to estimate associations.

**Results:**

Among 21,625 adults (representing ≈245 million U.S. adults), the mean age was 46.4 years. Low education was associated with 2.34 times higher odds of low CVH in males (95% CI: 1.72–3.20) and 1.83 times higher odds in females (95% CI: 1.38–2.43). Low income was linked to 2.79 times higher odds of low CVH in females (95% CI: 2.08–3.75). Unemployment was associated with 2.56 times higher odds of low CVH in males (95% CI: 1.91–3.44). Adults with ≥4 social risks had 7.14 times higher odds of low CVH in females and 6.05 in males. Mean LE8 scores declined from 68.0 to 60.9 in females and from 67.2 to 60.3 in males over the decade.

**Conclusion:**

Individual and cumulative social risks were strongly associated with lower CVH, with women bearing a greater burden. These findings highlight the need for policies that address social factors to reduce sex-based disparities and improve CVH.

**Figure:**
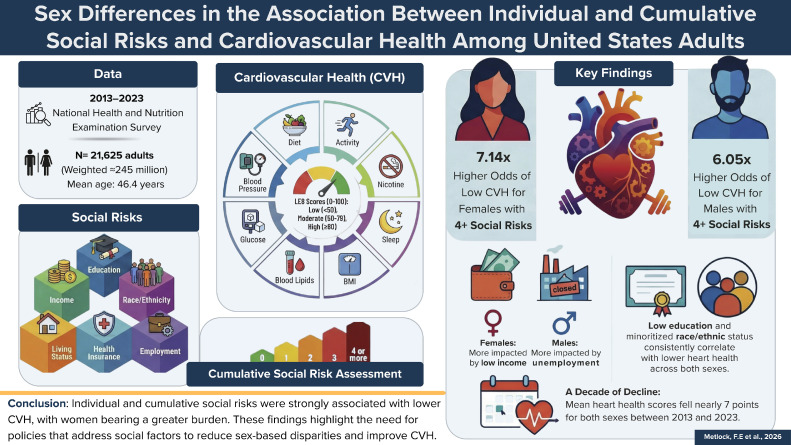
Central Illustration.

## 1 Introduction

Cardiovascular health (CVH) disparities persist in the United States, with notable differences between males and females. Males in the U.S. consistently demonstrate lower Life’s Essential 8 (LE8) scores than females, primarily due to poorer dietary habits, higher nicotine exposure, and less optimal blood pressure control [1]. Additionally, males tend to develop hypertension, coronary artery disease, and myocardial infarction at younger ages, whereas females experience a steeper increase in cardiovascular risk after menopause due to metabolic and hormonal changes [2,3]. Despite these known sex differences, the mechanisms underlying these disparities extend beyond biological factors to include broader social and environmental determinants that shape cardiovascular risk over the life course [4].

A growing body of evidence underscores the role of social determinants of health (SDoH) including income, education, employment, housing stability, and access to healthcare, in influencing cardiovascular outcomes [[4], [5], [6]]. Individuals experiencing multiple social disadvantages are more likely to develop hypertension, obesity, diabetes, and dyslipidemia, increasing their risk of cardiovascular events [7,8]. While the association between individual social risk factors and CVH has been well documented, fewer studies have examined whether these social risks impact males and females differently. Given the established sex differences in cardiovascular disease (CVD) incidence and progression, it is imperative to determine whether social disadvantage contributes to these disparities in distinct ways.

Social risks rarely occur in isolation, yet most research investigates single determinants rather than their cumulative impact. Factors such as food insecurity, financial strain, limited healthcare access, and neighborhood deprivation tend to cluster together, amplifying their adverse effects [9]. Emerging evidence suggests that cumulative disadvantage is a stronger predictor of cardiovascular outcomes than any single risk factor alone [6,10,11]. However, few studies have assessed cumulative social disadvantage in the context of LE8, a framework that evaluates diet, physical activity, nicotine exposure, sleep health, body mass index (BMI), blood pressure, blood glucose, and blood lipids [12].

To address these gaps, this study examines how cumulative social disadvantage, defined as the accumulation of multiple social risk factors, impacts CVH among U.S. adults stratified by sex using data from the National Health and Nutrition Examination Survey (NHANES). By assessing LE8 components across different levels of social risk burden, this study provides a comprehensive, sex-specific analysis of how social risks shape CVH outcomes. These findings can inform targeted public health interventions and policies aimed at reducing social disparities in CVH and advancing health equity across populations.

## 2 Methods

### 2.1 Study population

**Fig. 1:**
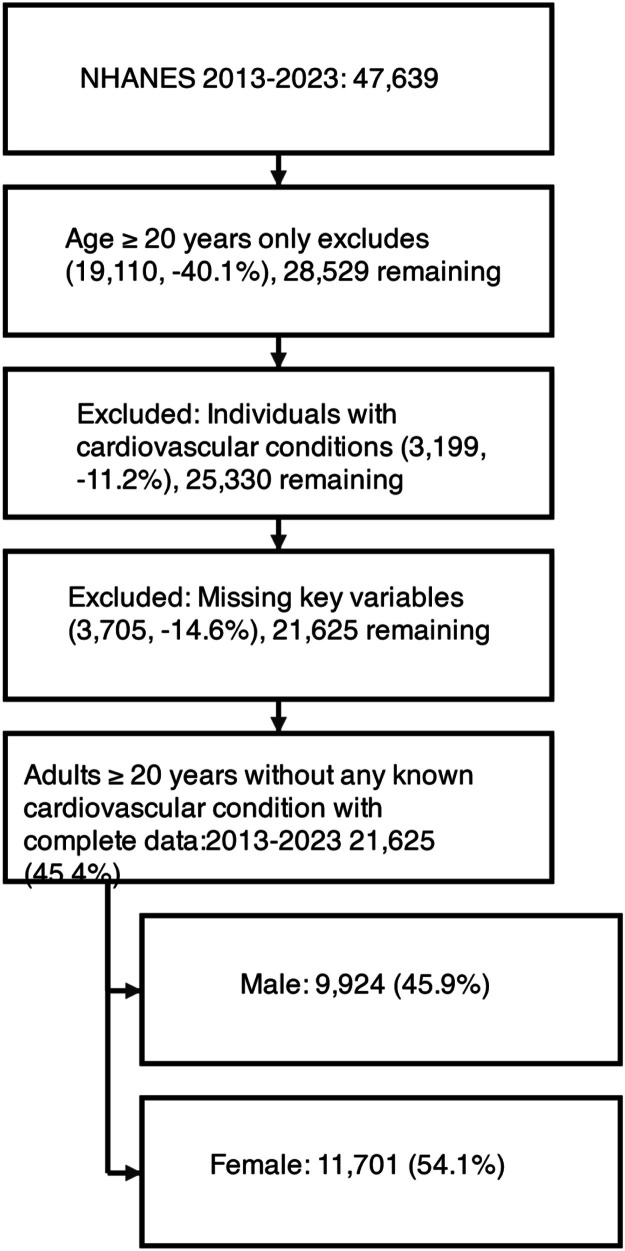
**Flowchart of Study Population Selection from NHANES 2013–2023** Flowchart depicting the selection of the study population from the National Health and Nutrition Examination Survey 2013–2023. The initial sample included all adults aged 20 years and older. Exclusions were applied for individuals with a self-reported history of cardiovascular disease (heart failure, stroke, myocardial infarction, or kidney disease) and those with missing data for key cardiovascular health (CVH) metrics or social determinants of health (SDoH) variables. The final analytic sample consisted of participants with complete data on all relevant measures.

This cross-sectional analysis utilized data from the NHANES for the years 2013–2023, a nationally representative survey that evaluates the health and nutritional status of the non-institutionalized U.S. population through interviews, physical examinations, and laboratory tests. This study sample included 47,639 adults aged 20 years and older. Participants were excluded if they were younger than 20 years (*n* = 19,110), had a self-reported history of CVDs, including heart failure, stroke, myocardial infarction, or kidney disease (*n* = 3199), or had missing data for key CVH metrics or SDoH variables (*n* = 3705). The final analytic sample consisted of 21,625 participants, including 9924 males and 11,701 females (Fig. 1**),** representing an estimated 245.4 million U.S. adults**.**

### 2.2 Social risk factors

Six social risk were examined as potential contributors to CVH disparities. Low family income was defined as a household income below the federal poverty level [13], as determined by the poverty-income ratio (PIR), with a PIR value of <1.0 indicating low income. Low educational attainment was classified as having a high school diploma or less, while participants with any college education or higher were categorized as having higher education. Minority race/ethnicity included participants who self-identified as Non-Hispanic Black, Hispanic, Non-Hispanic Asian, or other non-White racial/ethnic groups, with Non-Hispanic White participants serving as the reference group. Single-living status was determined based on marital or cohabitation status; participants who were widowed, divorced, separated, or never married were classified as single, while those who were married or living with a partner were considered cohabitating. Insurance status was dichotomized into insured or uninsured, where participants reporting any type of health insurance coverage, including private, Medicaid, or Medicare, were classified as insured, and those without coverage were classified as uninsured. Employment status was categorized as employed or unemployed based on participants’ self-reported work status at the time of the survey. Participants who were working part-time or full-time were classified as employed, while those who were unemployed, retired, disabled, or out of the workforce for other reasons were considered not employed. Each variable was coded consistent with prior studies examining the influence of social risk on CVH outcomes [11].

### 2.3 Life’s Essential 8

CVH was assessed using LE8, a metric developed by the American Heart Association that evaluates four health behaviors (diet, physical activity, nicotine exposure, and sleep health) and four health factors (BMI, blood pressure, blood glucose, and non-HDL cholesterol) [12]. Each metric was scored on a 0–100 scale, with higher scores indicating better CVH. Diet quality was assessed using adherence to the Dietary Approaches to Stop Hypertension (DASH) dietary pattern, which considers key components such as fruit and vegetable intake, whole grains, sodium, and fiber consumption, derived from a 24-hour dietary recall questionnaire. Physical activity was evaluated using the Computer-Assisted Personal Interviewing questionnaire, which measures self-reported engagement in moderate-to-vigorous physical activity over the past 30 days. Weekly minutes of moderate and vigorous activity were summed to determine total physical activity levels. Nicotine exposure was assessed based on self-reported current smoking status, smoking history, and exposure to secondhand smoke. Sleep health was determined by self-reported sleep duration. BMI was calculated from standardized weight (kg) and height (m²) measurements collected during physical examinations. Blood pressure was measured three times using a standardized mercury sphygmomanometer after participants rested quietly for five minutes in a seated position; the average of the second and third BP readings was used for analysis. Blood glucose levels were assessed using glycated hemoglobin concentrations, as NHANES does not collect fasting plasma glucose data on all participants. Non-HDL cholesterol was calculated from total cholesterol and HDL cholesterol. For each participant, individual metric scores were averaged to generate an overall LE8 score (0–100), which was then categorized as low (0–49), moderate (50–79), or high (80–100) CVH. **(Table S1.)**

### 2.4 Statistical analysis

Associations between social risk and CVH were assessed using multinomial logistic regression models. Each CVH metric, as well as the overall LE8 score, was modeled as a categorical variable with three levels: low, moderate, and high, using the high category as the reference group. Separate models were conducted for each individual CVH metric to evaluate the likelihood of low or moderate scores relative to high scores for each social risk factor, adjusting for the other primary social risk. Models were adjusted for age, sex, and the other social risk factors not serving as the primary exposure in a given model. To evaluate cumulative effects, the number of social risk experienced by each participant was summed up, creating categories of 0, 1, 2, 3, or ≥4 risks. Survey weighted multinomial logistic regression was then used to assess the association between cumulative social risk and CVH outcomes. Sex stratified analyses were performed to examine differences in these associations among male and female participants, with results presented separately by sex. Effect modification by sex was further evaluated by including interaction terms between cumulative social risk category and sex in survey weighted multinomial logistic regression models, and the joint significance of the interaction terms was assessed using an adjusted Wald test.

NHANES uses a complex, multistage probability sampling design with stratification, clustering, and oversampling to obtain a nationally representative sample of the noninstitutionalized United States population. All analyses incorporated MEC examination weights, strata (SDMVSTRA), and primary sampling units (SDMVPSU) to account for the complex survey design, with weights recalculated when combining survey cycles according to NHANES analytic guidelines. Because the LE8 categories are ordinal, we assessed the proportional odds assumption for ordinal logistic regression using Brant and Wolfe Gould tests. This assumption was violated for the overall LE8 score and all component outcomes (all *p* < 0.001); therefore, multinomial logistic regression was used to estimate category specific associations **(Table S2).** Regression results were expressed as relative risk ratios with 95 % confidence intervals.

## 3 Results

### 3.1 Sociodemographic characteristics

The weighted sample included 245 million U.S. adults, with an overall mean age of 46.4 years. Adults in the low LE8 category were older on average, particularly females (53.3 years), compared to those in the high LE8 category (40.1 years). Women represented 53.4 % of the sample. Non-Hispanic White adults were the most represented group across all LE8 categories, comprising 61 % of the low, 63 % of the moderate, and 67 % of the high LE8 group among females. Non-Hispanic Black adults made up 18 % of the low LE8 category for females and 15 % for males, with smaller proportions in the high category (6 % females, 7 % males). Marriage or cohabitation was more common in the high LE8 group (60 % females, 60 % males) than in the low group (47 % females, 57 % males). Educational attainment and income increased with better CVH. Nearly one in five adults in the low LE8 group had not completed high school, and financial hardship (PIR <1.0) was most prevalent in this group (25 % females, 21 % males). Employment rose steadily from the low to high LE8 groups, reaching 71 % among high LE8 females and 82 % among high LE8 males. Insurance coverage followed a similar trend, with 90 % of high LE8 females and 86 % of high LE8 males being insured. Table 1**.**

**Table 1:** Sociodemographic and Social Risks among NHANES Participants.

Characteristics	Total	Females (*N* = 11,701) 128,787,781			Males (*N* = 9924) 116,607,920		
		Life’s Essential 8 Scores					
		Low	Moderate	High	Low	Moderate	High
**Sample Size (N)**	21,625	2189	6911	2601	1770	6476	1678
**Population**	245,395,700	13,976,544	77,758,413	37,052,824	12,032,393	79,975,938	24,599,589
**Frequency (%) or mean ± SD**							
Age	46.4 ± (16.6)	53.47 ± (16.52)	50.75 ± (16.96)	40.55 ± (15.54)	51.26 ± (16.23)	49.67 ± (16.85)	41.74 ± (17.45)
Age, 20–50	100,184,842 (41)	8475,024 (61)	37,479,685 (48)	9192,237 (25)	5866,966 (49)	32,343,121 (40)	6827,809 (28)
Age, 51–85	145,210,859 (59)	5501,521 (39)	40,278,728 (52)	27,860,586 (75)	6165,427 (51)	47,632,817 (60)	17,771,779 (72)
**Race/ethnicity**							
Non-white status	88,624,116 (36)	5417,755 (39)	28,955,090 (37)	12,323,419 (33)	5203,886 (43)	28,426,993 (36)	8296,974 (34)
Mexican American	20,450,718 (8)	927,042 (7)	6285,387 (8)	2702,866 (7)	1142,182 (9)	7481,767 (9)	1911,475 (8)
Other Hispanic	17,304,495 (7)	912,868 (7)	5615,135 (7)	2691,565 (7)	946,778 (8)	5522,742 (7)	1615,407 (7)
Non-Hispanic White	156,771,584 (64)	8558,789 (61)	48,803,323 (63)	24,729,405 (67)	6828,507 (57)	51,548,945 (64)	16,302,615 (66)
Non-Hispanic Black	26,671,241 (11)	2558,098 (18)	10,058,182 (13)	2390,558 (6)	1816,336 (15)	8017,407 (10)	1830,660 (7)
Non-Hispanic Asian	14,387,799 (6)	243,495 (2)	3924,039 (5)	3645,456 (10)	451,657 (4)	3987,327 (5)	2135,825 (9)
Other Race – Multi-Racial	9809,862 (4)	776,251 (6)	3072,347 (4)	892,974 (2)	846,933 (7)	3417,750 (4)	803,607 (3)
**Marital Status**							
Married/cohabitating	145,662,004 (59)	6619,556 (47)	43,993,313 (57)	22,232,193 (60)	6863,770 (57)	51,246,573 (64)	14,706,600 (60)
Not married	99,733,696 (41)	7356,989 (53)	33,765,100 (43)	14,820,631 (40)	5168,623 (43)	28,729,366 (36)	9892,989 (40)
**Education**							
≥ High school graduate	218,068,038 (89)	11,812,469 (85)	68,548,335 (88)	35,033,698 (95)	9754,647 (81)	70,130,919 (88)	22,787,970 (93)
< High school graduate	27,327,662 (11)	2164,075 (15)	9210,077 (12)	2019,126 (5)	2277,746 (19)	9845,019 (12)	1811,618 (7)
**Family Poverty Income Ratio**							
≥ 1.00	211,937,379 (86)	10,547,935 (75)	65,631,937 (84)	33,273,078 (90)	9450,388 (79)	71,048,541 (89)	21,985,500 (89)
< 1.00	33,458,322 (14)	3428,609 (25)	12,126,476 (16)	3779,746 (10)	2582,005 (21)	8927,397 (11)	2614,089 (11)
**Insurance**							
Insured	212,427,247 (87)	12,283,372 (88)	68,538,494 (88)	33,495,222 (90)	9733,103 (81)	67,288,987 (84)	21,088,069 (86)
Uninsured	32,968,454 (13)	1693,172 (12)	9219,918 (12)	3557,602 (10)	2299,290 (19)	12,686,951 (16)	3511,520 (14)
**Employment**							
Employed	165,487,925 (67)	6440,206 (46)	45,867,031 (59)	26,353,569 (71)	7036,616 (58)	59,715,649 (75)	20,074,855 (82)
Unemployed	79,907,775 (33)	7536,338 (54)	31,891,381 (41)	10,699,255 (29)	4995,777 (42)	20,260,290 (25)	4524,734 (18)
**Life’s Essential 8 Scores**							
median (IQR)	69.38 (58.75- 80.00)	43.75 (38.12–46.88)	66.88 (59.38–72.50)	87.50 (83.12–91.25)	43.75 (38.12–46.88)	66.88 (60.00–73.12)	85.62 (82.50–90.00)

Life’s Essential 8 scores were categorized as low (0–49), moderate (50–79), or high (80–100).

Weighted analyses were conducted to account for the complex survey design of the National Health and Nutrition Examination Survey (NHANES).

### 3.2 Social risk factors by sex

**Fig. 2:**
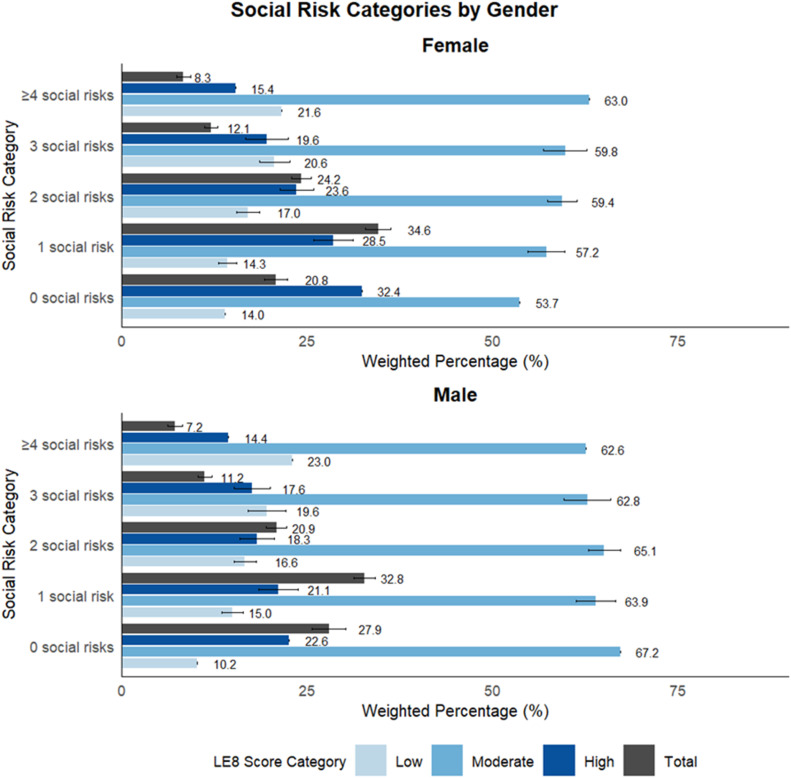
**Prevalence of Social Risk Factors by Life’s Essential 8 Score Categories** The distribution of social risk factors across Life’s Essential 8 cardiovascular health categories (low, moderate, and high) and the total sample. Social risk factors include low family income, low education, minority race/ethnicity, single-living status, uninsured status, and unemployment. Each bar represents the percentage of participants within each LE8 category who reported a given number of social risk factors (0, 1, 2, 3, or 4 or more). Weighted analyses were applied to ensure national representativeness.

Males were more likely to have zero social risk (27.9 %) compared to females (20.8 %). However, a greater proportion of females had one (34.6 %) and two (24.2 %) social risk compared to males (32.8 % and 20.9 %, respectively). The proportion of individuals with three social risk was slightly higher among females (12.1 %) than males (11.2 %). Additionally, a higher percentage of females (8.3 %) had four or more social risk compared to males (7.2 %). Fig. 2 presents the prevalence of social risk factors across low, moderate, and high LE8 categories, as well as in the total sample**.**

### 3.3 Association of social risk factors with life’s essential 8 components in females

As presented in Table 2, multiple social risk factors were linked to higher adjusted odds of scoring in the low or moderate LE8 categories across several CVH metrics among females.

**Table 2:** Adjusted Odds Ratios for Social Risk factors and Life's Essential 8 among US Females.

**Characteristics**	low family income	low education level	minority race/ethnic group	single-living status	uninsured	unemployed
Blood sugar						
Low (0–49)	**1.37** **(1.06–1.78)**	**1.52** **(1.22–1.90)**	**2.39** **(2.01–2.83)**	**1.24** **(1.05–1.47)**	0.79 (0.63–1.01)	**1.28** **(1.06–1.54)**
Moderate (50–79)	1.16 (0.97–1.38)	**1.29** **(1.06–1.57)**	**1.66** **(1.48–1.87)**	1.01 (0.89–1.13)	1.07 (0.89–1.28)	0.99 (0.86–1.14)
High (80–100)	*1.0*	*1.0*	*1.0*	*1.0*	*1.0*	*1.0*
Cholesterol						
Low (0–49)	**1.22** **(1.03–1.45)**	0.94 (0.78–1.14)	0.98 (0.88–1.09)	0.88 (0.77–1.00)	1.07 (0.90–1.28)	1.05 (0.92–1.19)
Moderate (50–79)	1.07 (0.92–1.24)	1.00 (0.83–1.20)	0.93 (0.82–1.06)	**0.72** **(0.64–0.82)**	1.11 (0.90–1.37)	**0.80** **(0.70–0.91)**
High (80–100)	*1.0*	*1.0*	*1.0*	*1.0*	*1.0*	*1.0*
Blood pressure`						
Low (0–49)	1.27 (0.99–1.63)	**1.34** **(1.08–1.66)**	**1.67** **(1.45–1.93)**	1.08 (0.94–1.24)	1.12 (0.91–1.37)	1.04 (0.90–1.20)
Moderate (50–79)	1.18 (0.99–1.41)	1.05 (0.85–1.31)	1.09 (0.96–1.24)	1.03 (0.90–1.18)	1.12 (0.89–1.42)	**0.77** **(0.66–0.90)**
High (80–100)	*1.0*	*1.0*	*1.0*	*1.0*	*1.0*	*1.0*
Smoking						
Low (0–49)	**2.00** **(1.69–2.37)**	**1.55** **(1.24–1.94)**	**0.38** **(0.32–0.45)**	**1.62** **(1.42–1.86)**	**1.81** **(1.50–2.17)**	**1.27** **(1.04–1.54)**
Moderate (50–79)	1.11 (0.91–1.34)	1.01 (0.83–1.22)	**0.45** **(0.39–0.53)**	1.05 (0.90–1.23)	0.94 (0.72–1.21)	**0.82** **(0.69–0.97)**
High (80–100)	*1.0*	*1.0*	*1.0*	*1.0*	*1.0*	*1.0*
Physical activity						
Low (0–49)	**1.16** **(1.02–1.32)**	**1.61** **(1.38–1.89)**	**1.35** **(1.21–1.52)**	**1.13** **(1.02–1.24)**	1.19 (1.00–1.42)	1.11 (0.99–1.24)
Moderate (50–79)	1.07 (0.77–1.50)	1.23 (0.93–1.63)	1.15 (0.91–1.47)	0.82 (0.66–1.01)	0.96 (0.69–1.33)	1.06 (0.83–1.34)
High (80–100)	*1.0*	*1.0*	*1.0*	*1.0*	*1.0*	*1.0*
Diet						
Low (0–49)	**1.30** **(1.16–1.46)**	0.89 (0.75–1.06)	**0.83** **(0.74–0.92)**	**1.12** **(1.01–1.23)**	1.00 (0.82–1.22)	1.01 (0.87–1.18)
Moderate (50–79)	1.05 (0.87–1.25)	0.84 (0.70–1.01)	**0.85** **(0.75–0.96)**	1.02 (0.88–1.19)	**1.25** **(1.01–1.56)**	0.93 (0.77–1.13)
High (80–100)	*1.0*	*1.0*	*1.0*	*1.0*	*1.0*	*1.0*
Sleep						
Low (0–49)	**1.58** **(1.25–2.00)**	1.18 (0.90–1.55)	**1.87** **(1.52–2.31)**	**1.56** **(1.32–1.83)**	1.12 (0.89–1.21)	0.98 (0.80–1.21)
Moderate (50–79)	**1.45** **(1.25–1.67)**	**1.39** **(1.16–1.67)**	**1.41** **(1.24–1.60)**	**1.38** **(1.23–1.55)**	1.06 (0.91–1.24)	1.16 (0.99–1.36)
High (80–100)	*1.0*	*1.0*	*1.0*	*1.0*	*1.0*	*1.0*
Body mass index						
Low (0–49)	**1.29** **(1.05–1.59)**	**1.25** **(1.05–1.49)**	**1.34** **(1.17–1.53)**	1.01 (0.89–1.16)	**1.32** **(1.10–1.60)**	**0.85** **(0.76–0.95)**
Moderate (50–79)	1.14 (0.93–1.40)	1.16 (0.95–1.41)	**1.18** **(1.03–1.34)**	**0.83** **(0.73–0.95)**	**1.27** **(1.05–1.53)**	0.96 (0.85–1.09)
High (80–100)	*1.0*	*1.0*	*1.0*	*1.0*	*1.0*	*1.0*

Population size: 128,787,781; Sample size: 11,701.

Social risk factors included low family income (poverty-income ratio <1.0), low education (high school diploma or less), minority race/ethnicity (Black, Hispanic, Asian, or other non-White groups), single-living status (widowed, divorced, separated, or never married), lack of insurance (no private, Medicaid, or Medicare coverage), and unemployment (not working, retired, or disabled). Cardiovascular health was assessed using Life’s Essential 8, which includes diet (DASH adherence), physical activity (moderate-to-vigorous activity minutes), nicotine exposure (current, former, or never smoking), sleep health (self-reported duration), BMI (poor: ≥30.0 kg/m², intermediate: 25.0–29.9 kg/m², ideal: <25.0 kg/m²), blood pressure (average of last two standardized readings), blood glucose (HbA1C levels), and non-HDL cholesterol. Individual scores (0–100) were averaged to categorize overall CVH as low (0–49), moderate (50–79), or high (80–100).

Low income and low education were most consistently associated with lower CVH. Women with low income had greater odds of scoring in the low category for smoking (aOR 2.00, 95 % CI 1.69–2.37), sleep (aOR 1.58, 95 % CI 1.25–2.00), and BMI (aOR 1.29, 95 % CI 1.05–1.59), compared to those with higher income. Similarly, low education was linked to higher odds of lower scores in blood sugar (aOR 1.52, 95 % CI 1.22–1.90), physical activity (aOR 1.61, 95 % CI 1.38–1.89), and BMI (aOR 1.25, 95 % CI 1.05–1.49). Associations with cholesterol and diet were closer to null. Among women from racial and ethnic minority groups, the odds of lower LE8 scores were particularly elevated for blood sugar (aOR 2.39, 95 % CI 2.01–2.83) and blood pressure (aOR 1.67, 95 % CI 1.45–1.93). However, this group had lower odds of low smoking scores (aOR 0.38, 95 % CI 0.32–0.45) and diet scores (aOR 0.83, 95 % CI 0.74–0.92).

Single-living status was linked to increased odds of being in the low category for smoking (aOR 1.62, 95 % CI 1.42–1.86), sleep (aOR 1.56, 95 % CI 1.32–1.83), and diet (aOR 1.12, 95 % CI 1.01–1.23), while it appeared protective for cholesterol (aOR 0.72, 95 % CI 0.64–0.82) and BMI (aOR 0.83, 95 % CI 0.73–0.95). Other associations were more modest. Among uninsured women, higher odds of low scores were observed for smoking (aOR 1.81, 95 % CI 1.50–2.17), physical activity (aOR 1.19, 95 % CI 1.00–1.42), and BMI (aOR 1.32, 95 % CI 1.10–1.60), while relationships with blood sugar, cholesterol, and sleep showed weaker or no associations. Unemployment was associated with elevated odds of low scores in blood sugar (aOR 1.28, 95 % CI 1.06–1.54) and smoking (aOR 1.27, 95 % CI 1.04–1.54). It was inversely associated with moderate blood pressure (aOR 0.77, 95 % CI 0.66–0.90) and cholesterol scores (aOR 0.80, 95 % CI 0.70–0.91).

### 3.4 Association of social risk factors with life’s essential 8 components in males

Table 3 shows adjusted associations between social risk factors and LE8 components among males. Low income was associated with higher odds of smoking (aOR 1.68, 95 % CI 1.43–1.98) and poor sleep (aOR 1.38, 95 % CI 1.08–1.76), but was not meaningfully associated with blood sugar, cholesterol, blood pressure, physical activity, diet, or BMI (aORs 0.90–1.15). Lower education was linked to elevated odds of poor blood sugar (aOR 1.56, 95 % CI 1.25–1.95), blood pressure (aOR 1.24, 95 % CI 1.04–1.47), smoking (aOR 1.79, 95 % CI 1.44–2.21), low physical activity (aOR 1.56, 95 % CI 1.29–1.88), and moderate sleep scores (aOR 1.28, 95 % CI 1.07–1.54), and lower odds of poor diet (aOR 0.84, 95 % CI 0.72–0.99). Racial and ethnic minority males had higher odds of poor blood sugar (aOR 2.20, 95 % CI 1.84–2.62), cholesterol (aOR 1.18, 95 % CI 1.03–1.34), blood pressure (aOR 1.45, 95 % CI 1.19–1.77), physical activity (aOR 1.30, 95 % CI 1.14–1.48), and sleep (aOR 1.71, 95 % CI 1.37–2.13), and lower odds of smoking (aOR 0.73, 95 % CI 0.63–0.87) and poor diet (aOR 0.81, 95 % CI 0.70–0.95). No association was observed for BMI (aOR 1.12, 95 % CI 0.95–1.33).

**Table 3:** Adjusted Odds Ratios for Social Risk^a^ factors and Life’s Essential 8^b^ among US Males.

**Characteristics**	low family income	low education level	minority race/ethnic group	single-living status	uninsured	unemployed
Blood sugar						
Low (0–49)	1.08 (0.82–1.33)	**1.56** **(1.25–1.95)**	**2.20** **(1.84–2.62)**	0.94 (0.76–1.17)	**0.74** **(0.56–0.96)**	**1.42** **(1.14–1.76)**
Moderate (50–79)	1.17 (0.96–1.42)	**1.25** **(1.07–1.45)**	**1.63** **(1.42–1.87)**	0.85 (0.72–0.99)	**0.79** **(0.67–0.94)**	0.92 (0.78–1.08)
High (80–100)	*1.0*	*1.0*	*1.0*	*1.0*	*1.0*	*1.0*
Cholesterol						
Low (0–49)	0.94 (0.78–1.12)	1.16 (0.97–1.39)	**1.18** **(1.03–1.34)**	**0.72** **(0.62–0.82)**	1.12 (0.94–1.33)	**0.72** **(0.62–0.84)**
Moderate (50–79)	0.91 (0.75–1.11)	1.17 (0.98–1.39)	1.05 (0.93–1.20)	**0.70** **(0.59–0.84)**	1.19 (0.99–1.43)	**0.69** **(0.56–0.86)**
High (80–100)	*1.0*	*1.0*	*1.0*	*1.0*	*1.0*	*1.0*
Blood pressure						
Low (0–49)	1.13 (0.88–1.46)	**1.24** **(1.04–1.47)**	**1.45** **(1.19–1.77)**	1.14 (0.98–1.34)	0.93 (0.72–1.20)	0.88 **(**0.71–1.10)
Moderate (50–79)	0.96 (0.77–1.20)	1.19 (1.00–1.42)	1.08 (0.94–1.35)	1.05 (0.91–1.22)	1.05 (0.87–1.25)	0.85 (0.72–1.00)
High (80–100)	*1.0*	*1.0*	*1.0*	*1.0*	*1.0*	*1.0*
Smoking						
Low (0–49)	**1.68** **(1.43–1.98)**	**1.79** **(1.44–2.21)**	**0.73** **(0.63–0.86)**	**1.65** **(1.41–1.94)**	**2.07** **(1.71–2.50)**	**1.36** **(1.14–1.61)**
Moderate (50–79)	0.96 (0.77–1.20)	**1.38** **(1.16–1.64**)	**0.75** **(0.65–0.86**)	0.93 (0.76–1.13)	1.13 (0.89–1.43)	1.08 (0.91–1.29)
High (80–100)	*1.0*	*1.0*	*1.0*	*1.0*	*1.0*	*1.0*
Physical activity						
Low (0–49)	1.15 (0.95–1.40)	**1.56** **(1.29–1.88)**	**1.30** **(1.14–1.48)**	1.02 (0.86–1.21)	1.02 (0.83–1.24)	**1.46** **(1.23–1.72**)
Moderate (50–79)	0.93 (0.59–1.46)	1.08 (0.71–1.66)	0.95 (0.72–1.25)	0.81 (0.60–1.11)	0.92 (0.60–1.41)	1.11 (0.74–1.66)
High (80–100)	*1.0*	*1.0*	*1.0*	*1.0*	*1.0*	*1.0*
Diet						
Low (0–49)	1.14 (0.94–1.38)	**0.84** **(0.72–0.99)**	**0.81** **(0.70–0.95)**	1.02 (0.87–1.19)	1.14 (0.95–1.35)	**1.34** **(1.16–1.56**)
Moderate (50–79)	0.93 (0.72–1.22)	0.90 (0.71–1.14)	**0.76** **(0.64–0.91)**	0.88 (0.71–1.08)	1.08 (0.85–1.38)	1.16 (0.95–1.43)
High (80–100)	*1.0*	*1.0*	*1.0*	*1.0*	*1.0*	*1.0*
Sleep						
Low (0–49)	**1.38** **(1.08–1.76)**	0.94 (0.73–1.21)	**1.71** **(1.37–2.13)**	**1.46** **(1.15–1.85**)	1.23 (0.94–1.60)	0.98 (0.78–1.23)
Moderate (50–79)	**1.24** **(1.04–1.49)**	**1.28** **(1.07–1.54)**	**1.22** **(1.07–1.39)**	**1.29** **(1.12–1.48)**	0.99 (0.82–1.19)	1.09 (0.92–1.28)
High (80–100)	*1.0*	*1.0*	*1.0*	*1.0*	*1.0*	*1.0*
High body mass index						
Low (0–49)	0.90 (0.75–1.06)	0.91 (0.75–1.11)	1.12 (0.95–1.33)	**0.64** **(0.54–0.75)**	0.81 (0.63–1.05)	0.91 (0.76–1.09)
Moderate (50–79)	0.83 (0.65–1.03)	0.98 (0.81–1.18)	1.13 (0.97–1.32)	**0.63** **(0.53–0.75)**	0.96 (0.77–1.19)	**0.79** **(0.68–0.92)**
High (80–100)	*1.0*	*1.0*	*1.0*	*1.0*	*1.0*	*1.0*

Population size: 116,607,920; Sample size: 9924.

a Social risks defined as: family income (poverty-income ratio <1.0), low education (high school diploma or less), minority race/ethnicity (Black, Hispanic, Asian, or other non-White groups), single-living status (widowed, divorced, separated, or never married), lack of insurance (no private, Medicaid, or Medicare coverage), and unemployment (not working, retired, or disabled). ^b^Life’s Essential 8 defined as: diet (DASH adherence), physical activity (moderate-to-vigorous activity minutes), nicotine exposure (current, former, or never smoking), sleep health (self-reported duration), BMI (poor: ≥30.0 kg/m², intermediate: 25.0–29.9 kg/m², ideal: <25.0 kg/m²), blood pressure (average of last two standardized readings), blood glucose (HbA1C levels), and non-HDL cholesterol. Individual scores (0–100) were averaged to categorize overall CVH as low (0–49), moderate (50–79), or high (80–100). Adjusted for age, gender, and social risks.

Single-living males had higher odds of smoking (aOR 1.65, 95 % CI 1.41–1.94) and poor sleep (aOR 1.46, 95 % CI 1.15–1.85), and lower odds of high cholesterol (aOR 0.72, 95 % CI 0.62–0.82) and high BMI (aOR 0.64, 95 % CI 0.54–0.75). Associations for other components were minimal (aORs 0.95–1.14). Uninsured males were more likely to smoke (aOR 2.07, 95 % CI 1.71–2.50) and had slightly lower odds of poor blood sugar (aOR 0.74, 95 % CI 0.56–0.96). Remaining associations for cholesterol, blood pressure, physical activity, diet, sleep, and BMI were small and inconsistent (aORs 0.81–1.23). Unemployment was associated with higher odds of poor blood sugar (aOR 1.42, 95 % CI 1.14–1.76), smoking (aOR 1.36, 95 % CI 1.14–1.61), low physical activity (aOR 1.46, 95 % CI 1.23–1.72), and poor diet (aOR 1.34, 95 % CI 1.16–1.56), as well as lower odds of moderate BMI (aOR 0.72, 95 % CI 0.68–0.92). Other metrics showed limited variation.

### 3.5 Cumulative social risks

**Fig. 3:**
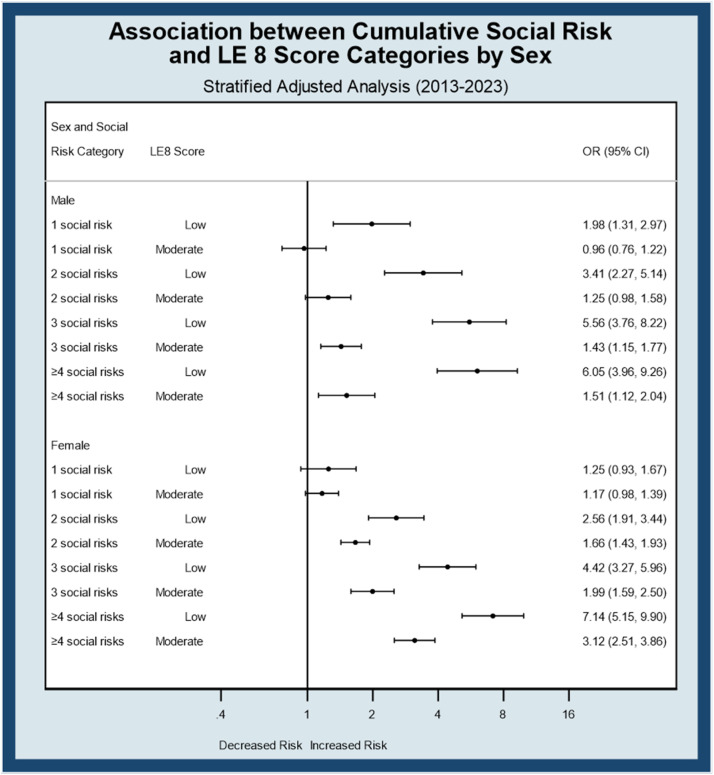
**Association Between Cumulative Social Risk and Life’s Essential 8 Score Categories by Sex** The relationship between cumulative social risk and Life’s Essential 8 score categories by sex. Cumulative social risk was determined by summing the total number of social risk factors (low family income, low education, minority race/ethnicity, single-living status, uninsured status, and unemployment), categorized as 0, 1, 2, 3, or 4 or more risks.

A clear stepwise relationship was observed between cumulative social risk and poorer CVH, with patterns more pronounced in females than males. Males with four or more social risk factors had nearly six times higher odds of low LE8 scores (aOR 6.05, 95 % CI 3.96–9.26) compared to those with no social risk, while females with four or more social risks had over seven times higher odds (aOR 7.14, 95 % CI 5.15–9.90). Among males, the odds of low CVH increased consistently with each additional social risk factor, beginning with one risk (aOR 1.98, 95 % CI 1.31–2.97) and rising across higher levels. Among females, the effect of cumulative social risk was particularly strong, with a twofold increase in the odds of low LE8 scores at two risks (aOR 2.56, 95 % CI 1.91–3.44) and more than fourfold at three risks (aOR 4.42, 95 % CI 3.27–5.96). Cumulative social risk was also associated with moderate LE8 scores, though the magnitude of associations was smaller. Males with four or more risks had 1.51 times higher odds (aOR 1.51, 95 % CI 1.12–2.04) of having moderate CVH compared to those with no risks, and females showed an even stronger pattern (aOR 3.12, 95 % CI 2.51–3.86). Formal interaction testing supported effect modification by sex in the association between cumulative social risk and LE8 category (adjusted Wald *F* = 4.17, *p* = 0.0005). Fig. 3**.**

### 3.6 Trends in social risk and life’s essential 8 scores by sex

**Fig. 4:**
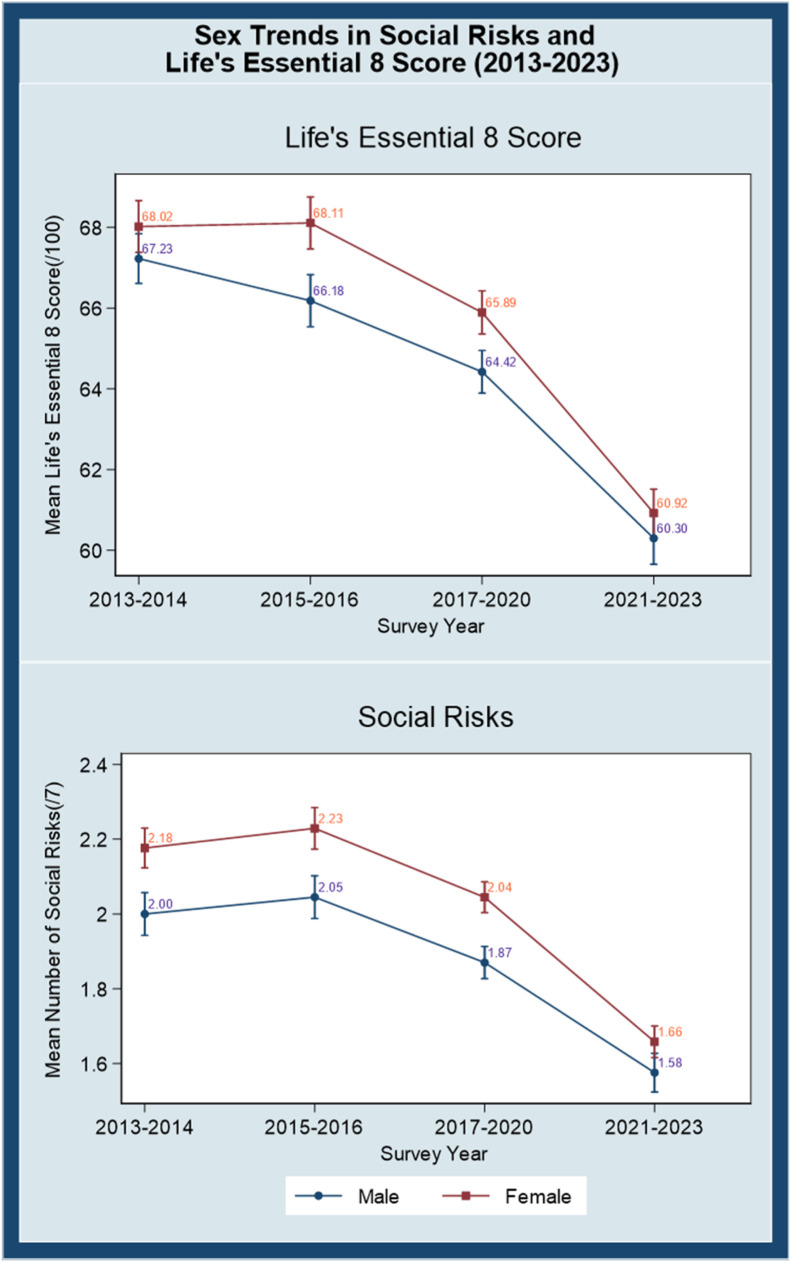
**Trends in Life’s Essential 8 Scores and Social Risk by Sex (2013–2023)** Trends in mean Life’s Essential 8 (LE8) scores and the average number of social risk factors among U.S. adults from 2013 to 2023, stratified by sex. LE8 scores (ranging from 0 to 100) reflect overall cardiovascular health (CVH), with higher scores indicating better CVH. Social risk factors (low family income, low education, minority race/ethnicity, single-living status, uninsured status, and unemployment) were assessed as a cumulative count, with a maximum possible score of six. The figure shows a decline in LE8 scores over time for both males and females, with a concurrent reduction in social risk burden. Weighted analyses were applied for national representativeness.

Over the past decade, trends in LE8 scores and social risk factors have shown a concerning decline in CVH among both males and females, despite a modest reduction in social risks. Among females, the mean LE8 score decreased from 68.12 in 2013–2014 to 61.25 in 2021–2023, while males experienced a similar decline from 67.30 to 60.55 over the same period, with both groups showing the most significant drop after 2017. Concurrently, the number of social risk (out of six possible) also declined, with females averaging 2.18 social risks in 2013–2014 and 1.66 in 2021–2023, while males dropped from 2.00 to 1.58. Fig. 4**.**

## 4 Discussion

Among a nationally representative sample of U.S. adults, several individual social risks, including low educational attainment, minoritized racial and ethnic status, low income, single-living status, lack of insurance, and unemployment, were associated with poorer CVH, with patterns varying by sex. Females were more adversely affected by low income, lack of insurance, and single-living status, while unemployment had stronger associations among males. These findings suggest that individual social risk factors may influence CVH through different pathways across sexes, particularly for economic and access-related stressors. Cumulative social risk showed a clear stepwise association with CVH, where each additional risk was associated with lower odds of achieving moderate or high LE8 scores. Adults with four or more social risks had the highest odds of low CVH, with more pronounced effects among females than males. Formal interaction testing further supported sex differences in the association between cumulative social risk and CVH, reinforcing the importance of sex-disaggregated analyses when examining how social disadvantage shapes CVH. Although some social risks have declined over time, CVH outcomes have worsened, pointing to the need for renewed focus on social and structural contributors to poor cardiovascular outcomes.

Low education was consistently associated with lower LE8 scores in both sexes and affected multiple CVH metrics, with more risks concentrated among males. These results mirror findings from Williams et al., who also reported lower LE8 scores among adults with limited education, especially females [14]. Education influences CVH through health literacy, access to resources, and use of preventive care [15]. Its strong connection to economic opportunity suggests that income may mediate this relationship. In our study, low income was linked to lower LE8 scores across metrics, with stronger effects among females, particularly for blood sugar, cholesterol, smoking, sleep, and BMI. These results align with prior studies showing that financial strain worsens CVH outcomes, especially among economically disadvantaged women [14]. Employment and insurance status further shape CVH by influencing access to care and financial stability [16]. Unemployment was associated with lower LE8 scores, particularly among males, aligning with Williams et al., who found poorer CVH among unemployed men [14]. Similarly, uninsured females in our study had higher odds of poor CVH metrics, including smoking, physical inactivity, and elevated BMI. These findings reinforce the importance of employment-based benefits in the U.S. and highlight the vulnerabilities created when insurance or income is unstable. Racial and ethnic minority status was also associated with poorer LE8 scores across several CVH metrics. Our findings build on research by Shah et al., who identified social and psychosocial factors as drivers of racial and ethnic disparities in CVH, including birthplace and educational background [17]. Williams et al. found that socioeconomic advantages do not benefit racial and ethnic minorities to the same extent as non-Hispanic White adults [18]. Structural racism and persistent inequities in healthcare access may limit the effectiveness of traditional interventions for these populations, underscoring the need for strategies that directly confront systemic barriers [4]. Living alone was another consistent predictor of poorer CVH outcomes in both sexes, particularly for blood sugar, smoking, physical activity, diet, and sleep. These associations may reflect reduced social support or increased stress. Although past research has shown that the quality of relationships may matter more than marital status alone, our findings align with broader literature linking social isolation to higher cardiovascular risk [[19], [20], [21]]. This highlights the potential value of interventions aimed at increasing social connectedness and support for those living alone.

Cumulative social risk emerged as one of the strongest predictors of poor CVH, particularly among females. Those with four or more social risks had significantly lower odds of achieving favorable LE8 scores. These findings align with Caleyachetty et al., who reported that individuals with three or more risks were 81 % less likely to achieve ideal CVH [11]. Our results extend this evidence by using LE8 and showing recent sex-specific patterns. The HeartSCORE study also supports this relationship, showing that multiple risks including economic strain and racial/ethnic minoritized status, contribute to poor CVH, with psychosocial stress acting as a mediator [22]. While some social risks have declined modestly, LE8 scores have continued to drop, particularly after 2017, suggesting that other unmeasured factors such as psychological distress may also contribute [23]. Given the strong and cumulative impact of social risk on CVH, healthcare and public health strategies must move upstream to integrate social context into routine care [24]. Screening for social risk in clinical settings can help identify individuals at higher risk and connect them with community-based support. Sex-specific interventions such as childcare subsidies, transportation access, and flexible work supports may help reduce the barriers faced by women, while efforts to strengthen job stability and increase wages could improve outcomes among men. Broader policies focused on employment, housing, education, and healthcare access are also needed to address the root causes of CVH disparities. Environmental and neighborhood factors, such as access to green space or exposure to pollution, may further compound these risks and warrant inclusion in future research [25]. Ultimately, improving CVH requires coordinated action across clinical, policy, and community domains. Public health systems must broaden their focus beyond individual behavior to include the underlying social and structural factors that shape health outcomes. Embedding social risk assessment into clinical workflows and scaling community-based solutions can help reduce inequities and advance CVH equity at the population level.

## 5 Limitations

This study has several limitations. First, its cross-sectional design limits causal interpretation; longitudinal data are needed to assess long-term effects. Second, social risk measures were self-reported, which may introduce recall bias and misclassification. Key risks such as financial strain and job insecurity may have been underrepresented. Third, our cumulative social risk measure was restricted to indicators that were available and consistently measured across NHANES survey cycles, which limited our ability to capture the full breadth and depth of social risk. Fourth, while LE8 provides a comprehensive measure of CVH, it relies on self-reported diet and physical activity and does not capture psychosocial stressors like discrimination or social support. Fifth, our cumulative social risk score weighted all risks equally, which may overlook differences in their impact. A weighted approach could offer greater precision. Lastly, NHANES excludes institutionalized and undocumented individuals, likely underestimating social risk burdens in the most vulnerable groups. Despite these limitations, the study offers important insights into how cumulative social risk shapes CVH across sexes and highlights the need for targeted policy solutions.

## 6 Conclusion

This study reinforces the link between cumulative social risk and poor CVH, with women experiencing greater vulnerability. Findings suggest that social factors, not just biological differences, shape sex disparities in cardiovascular outcomes. Addressing these risks through routine screening, community referrals, and policies that support financial and healthcare access may help reduce inequities. Future efforts should prioritize integrating social risk measures into care and exploring long-term impacts to guide more equitable interventions.

## Acknowledgements

Funding: FEM is supported by the National Institute of Nursing Research of the National Institutes of Health under award Number T32NR020315 and the 10.13039/100000968American Heart Association under award number 24PRE12438460. YCM and CRD are supported by the American Heart Association Health Equity Research Network on the Prevention of Hypertension (Grant number: 882415) and 10.13039/100006545National Institute on Minority Health and Health Disparities (Grant number: P50MD017348–818). OO is supported by the American Heart Association Healthcare by Food Initiative (24FIM1264121), American Heart Association Food is Medicine Planning Award (25FIMPG1466841), American Heart Association Implementation Science Award (25ISA1453143), American Heart Association Career Development Award (25CDA1453103), U24 NIDDK BRIDGES-COURAGE (Creating Opportunities for Unparalled Researchers to Achieve Growth and Excellence) program (U24DK132733), and the Mid-Atlantic Center for Cardiometabolic Health (MACCH).

## CRediT authorship contribution statement

**Faith E. Metlock:** Writing – review & editing, Writing – original draft, Visualization, Validation, Methodology, Investigation, Formal analysis, Conceptualization. **Ketum Ateh Stanislas:** Writing – review & editing, Methodology, Formal analysis. **Oluwabunmi Ogungbe:** Writing – review & editing. **Biruk Teshome:** Writing – review & editing, Methodology. **Thomas Hinneh:** Writing – review & editing. **Ruth-Alma Turkson-Ocran:** Writing – review & editing. **Cheryl R. Himmelfarb:** Writing – review & editing. **Garima Sharma:** Writing – review & editing, Conceptualization. **Yvonne Commodore-Mensah:** Writing – review & editing, Supervision, Methodology, Conceptualization.

## Declaration of competing interest

The authors declare that they have no known competing financial interests or personal relationships that could have appeared to influence the work reported in this paper.

## Appendix Supplementary materials

Image, application 1
