# Supplementary material for: Sex differences in the association between cumulative social risk and life’s essential 8 cardiovascular health scores in U.S. adults
Source: Am J Prev Cardiol. 2026 Mar 17;28:101566. doi: 10.1016/j.ajpc.2026.101566 (PMC13325737; doi:10.1016/j.ajpc.2026.101566)
Supplement: Supplementary file 1 [file mmc1.docx]

**Supplemental Files**

| **Table S1. Life’s Essential 8 Scoring** | |
| --- | --- |
| **Life’s Essential 8 Metric** | **Quantification of Metric** |
| **Diet** | **Metric**: Quantiles of DASH-style diet adherence  **Scoring (Population):**  Points Quantile  100 ≥95^th^ %ile (top/ideal diet)  80 75^th^ – 94^th^ %ile  50 50^th^ – 74^th^ %ile  25 25^th^ – 49^th^ %ile  0 1^st^ – 24^th^ %ile (bottom/least ideal quartile |
| **Physical activity** | **Metric:** Minutes of moderate (or greater) intensity activity per week  **Scoring:**  Points Minutes  100 ≥150  90 120 – 149  80 90 – 119  60 60 – 89  40 30 – 59  20 1 – 29  0 0 |
| **Nicotine exposure** | **Metric:** Combustible tobacco use and/or inhaled NDS use; or secondhand smoke exposure  **Scoring (2013-2020):**  Points Status  100 Never smoker  75 Former smoker, quit ≥5 yrs  50 Former smoker, quit 1 - <5 yrs  25 Former smoker, quit <1 year, or currently using inhaled NDS  0 Current smoker  Subtract 20 points (unless score is 0) for living with active indoor smoker in home  **Scoring (2021-2023):**  Points Status  100 Never smoker  50 Smoked 100 cigarettes in lifetime but not currently a smoker  0 Current smoker |
| **Sleep health** | **Metric:** Average hours of sleep per night  **Scoring:**  Points Level  100 7 – <9  90 9 - <10  70 6 - <7  40 5 - <6 or ≥10  20 4 - <5  0 <4 |
| **Body mass index** | **Metric:** Body mass index (kg/m^2^)  **Scoring:**  Points Level  100 <25  70 25.0 – 29.9  30 30.0 – 34.9  15 35.0 – 39.9  0 ≥40.0 |
| **Blood sugar** | **Metric:** Hemoglobin A1c (%)  **Scoring:**  Points Level  100 No history of diabetes and FBG <100 (or HbA1c < 5.7)  60 No diabetes and FBG 100 – 125 (or HbA1c 5.7-6.4) (Pre-diabetes)  40 Diabetes with HbA1c 6.5 <7.0  30 Diabetes with HbA1c 7.0 – 7.9  20 Diabetes with HbA1c 8.0 – 8.9  10 Diabetes with Hb A1c 9.0 – 9.9  0 Diabetes with HbA1c ≥10.0 |
| **Blood pressure** | **Metric:** Average systolic and Diastolic blood pressure values  **Scoring:**  Points Level  100 <120/<80 (Optimal)  75 120-129/<80 (Elevated)  50 130-139 or 80-89 (Stage I HTN)  25 140-159 or 90-99  0 ≥160 or ≥100  Subtract 20 points if treated level |
| **Cholesterol** | **Metric:** Non-HDL-cholesterol (mg/dL)  **Scoring:**  Points Level  100 <130  60 130 – 159  40 160 – 189  20 190 – 219  0 ≥220  If drug-treated level, subtract 20 points |


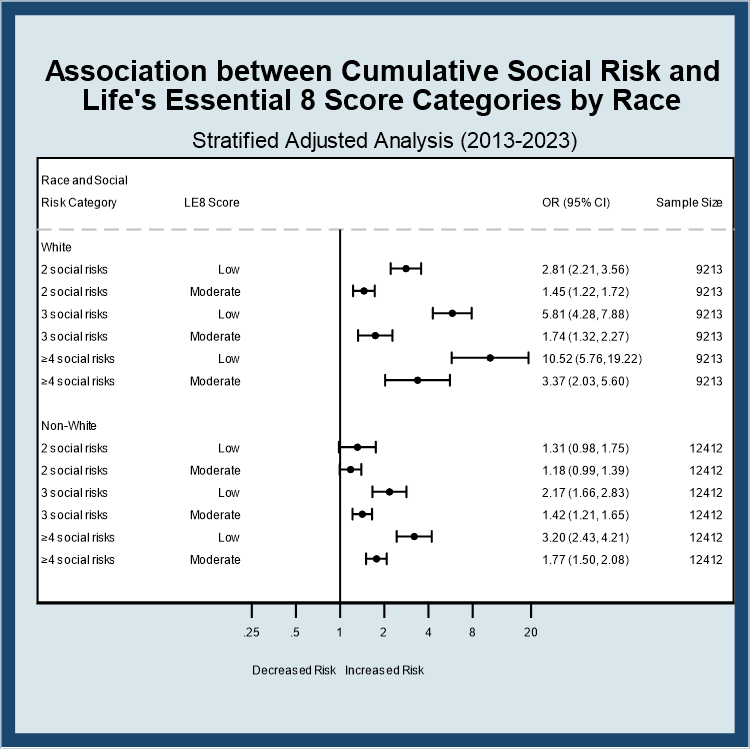


**Figure: Racial Trends in Cumulative social risks and LE8 scores by race**


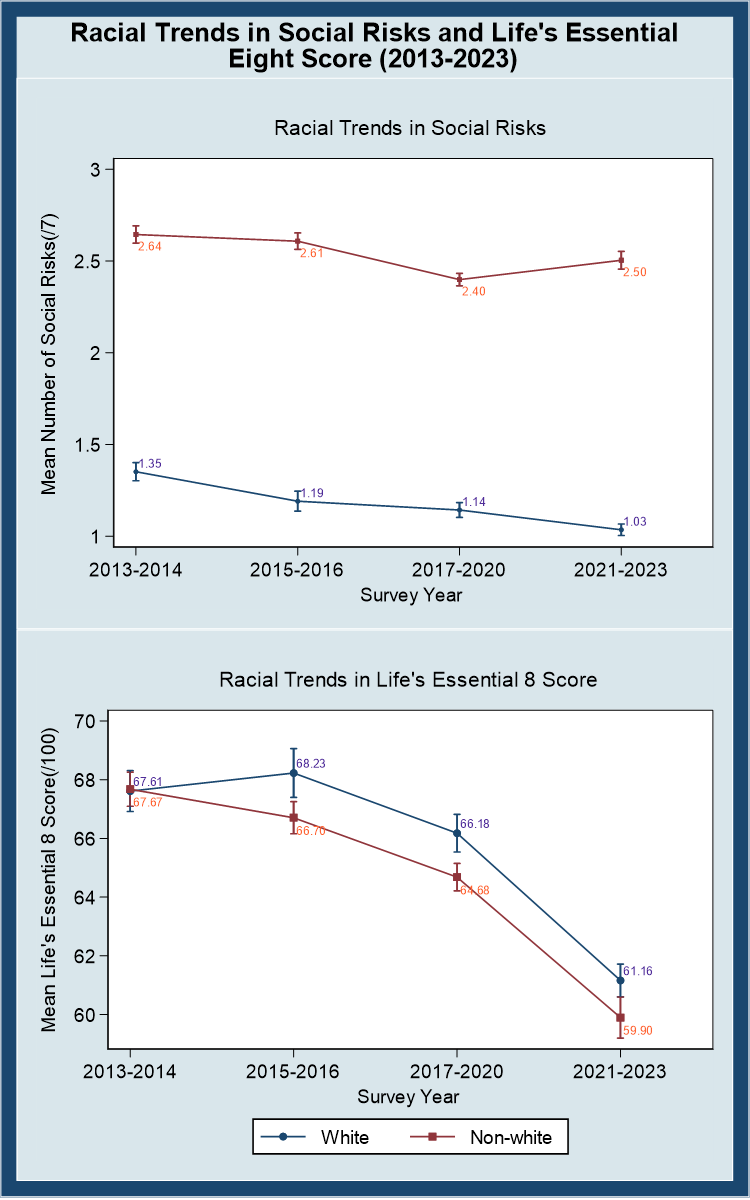


**Figure. Cumulative social risks and mean LE8 scores**


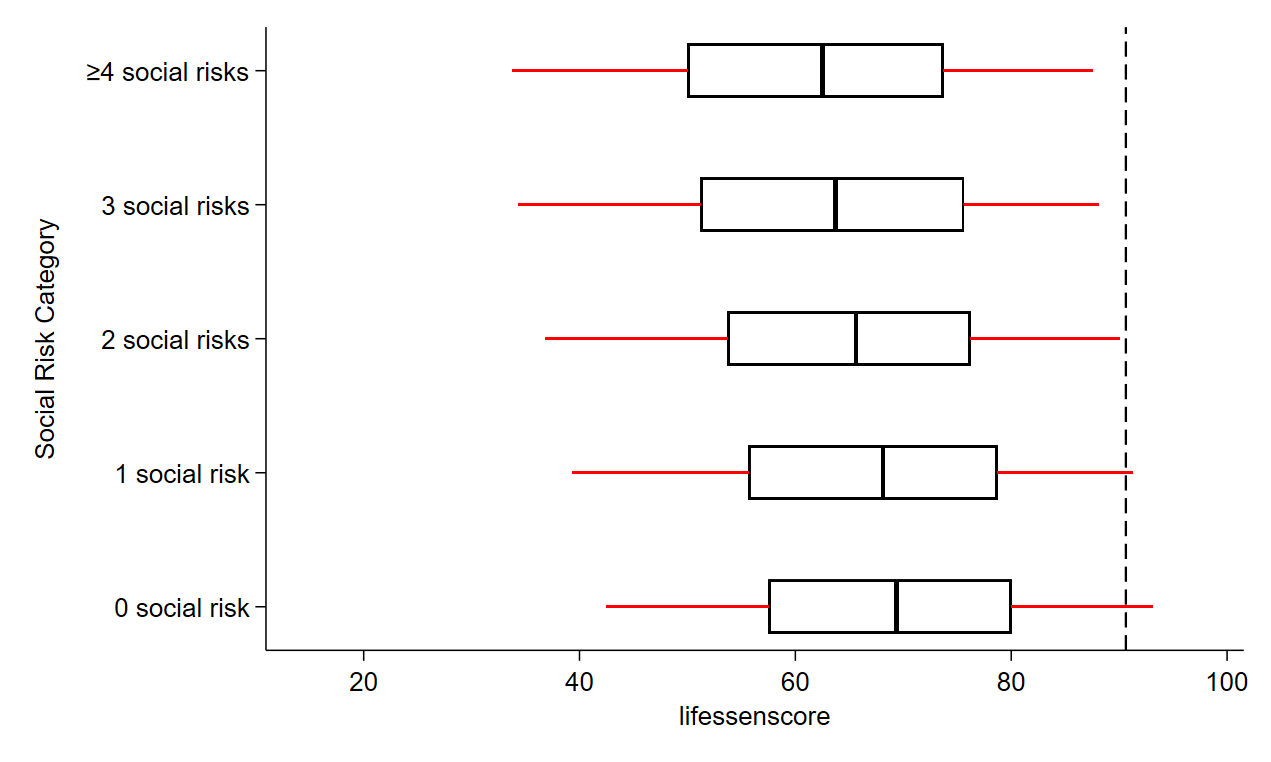


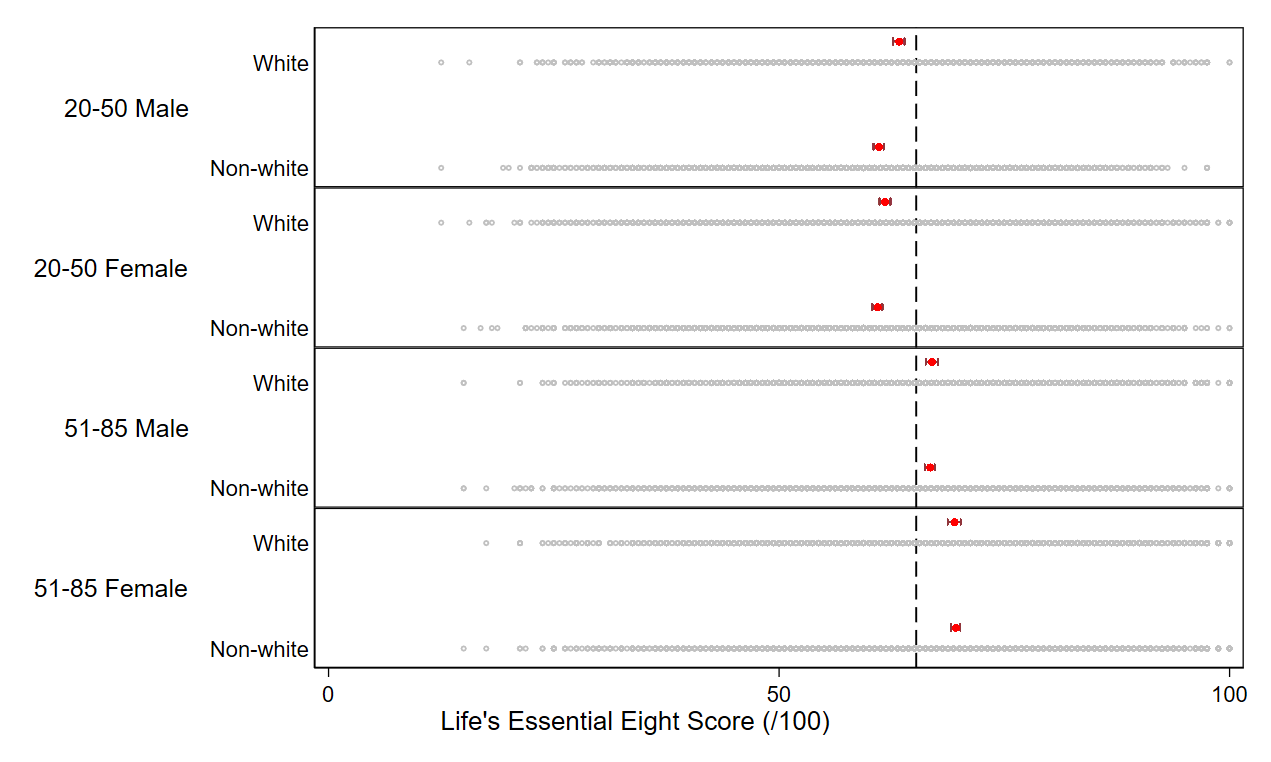


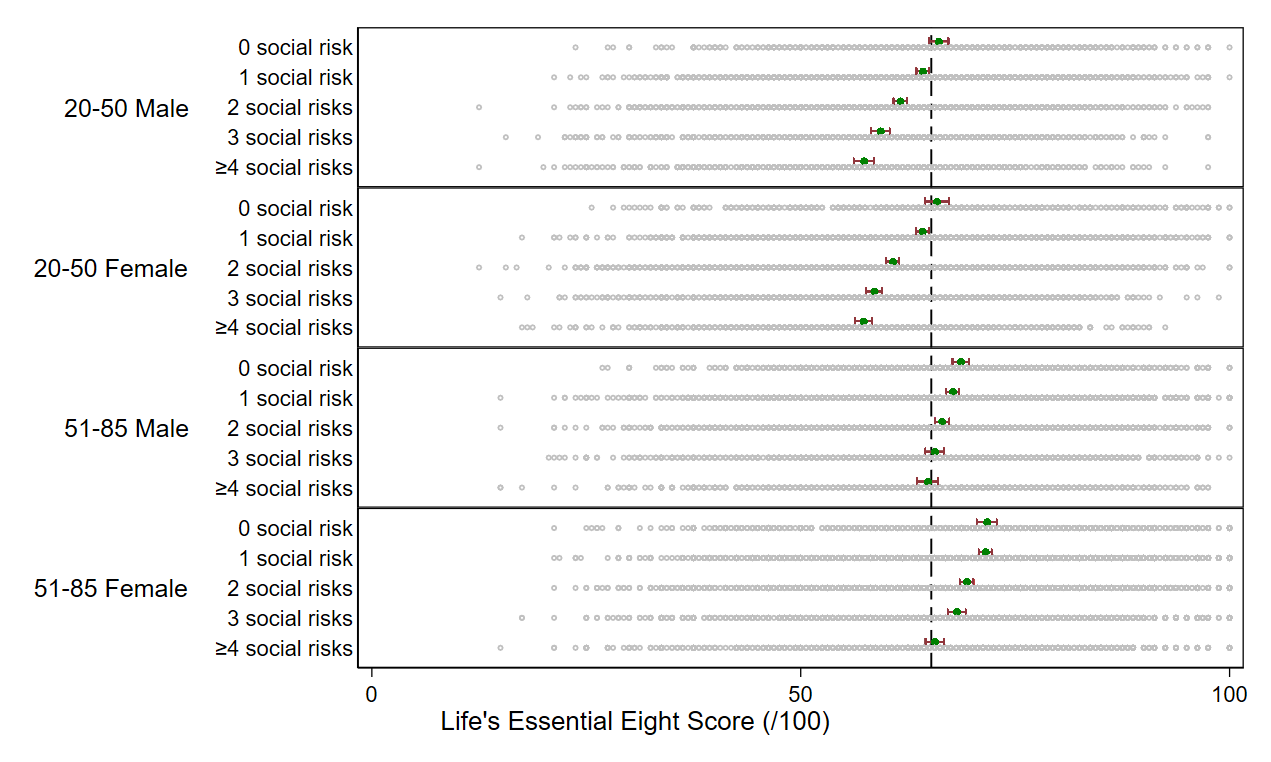


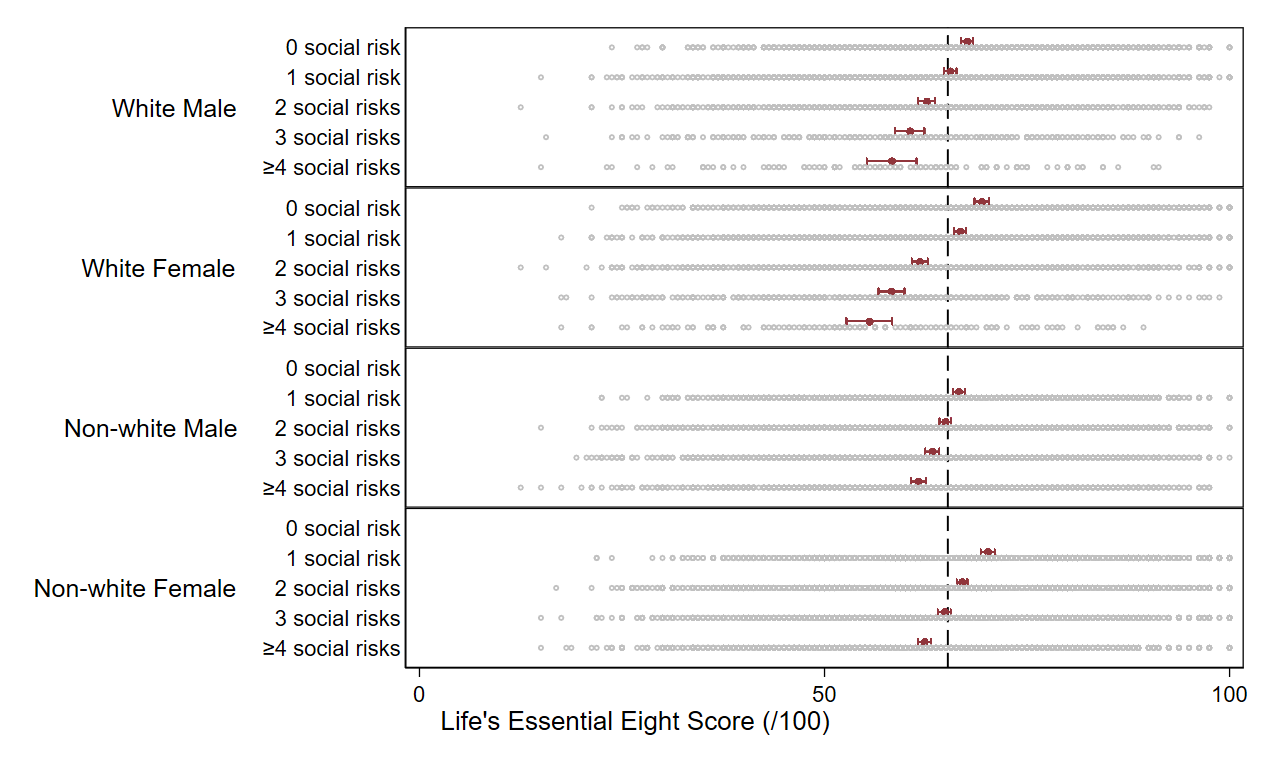


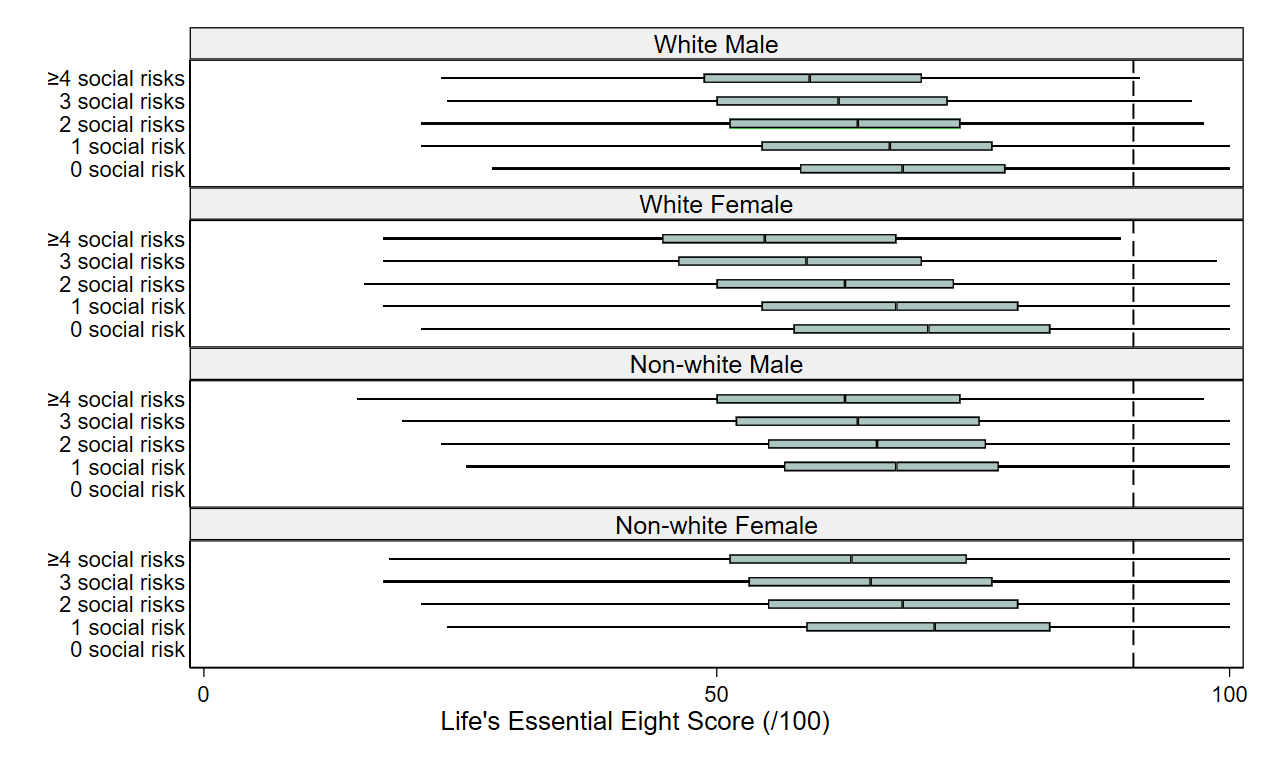


**Table S2. Tests of the Proportional Odds Assumption for Ordinal Models**

| **Outcome** | **Brant χ² (df=7)** | **P-value** | **Wolfe–Gould χ² (df=7)** | **P-value** |
| --- | --- | --- | --- | --- |
| LE8 Total Score | 292.2 | <0.001 | 276.3 | <0.001 |
| Physical Activity | 47.2 | <0.001 | 40.4 | <0.001 |
| Blood Pressure | 105.9 | <0.001 | 97.6 | <0.001 |
| Glucose | 608.4 | <0.001 | 594.1 | <0.001 |
| Lipids | 93.8 | <0.001 | 95.7 | <0.001 |
| BMI | 239.2 | <0.001 | 220.2 | <0.001 |
| Sleep | 358.4 | <0.001 | 347.3 | <0.001 |
| Smoking | 1519.0 | <0.001 | 1552.0 | <0.001 |
| Diet (DASH) | 72.7 | <0.001 | 76.5 | <0.001 |

***Note:****Tests conducted using the oparallel command in Stata 18 following unweighted ordinal logistic regression. All tests reject the proportional odds (parallel lines) assumption at p < 0.001, justifying the use of multinomial logistic regression.*

| **Table S3. Association Between Cumulative Social Risks^a^ and Life’s Essential 8^b^ in US Adults (N= 21,625))** | | | | | |
| --- | --- | --- | --- | --- | --- |
| **Number of social risks factors^c^** | | | | | |
| **LE8 Scores**  **(mean)** | **0** | **1** | **2** | **3** | **≥ 4** |
| **Adjusted Odds Ratio, 95% CI** | | | | | |
| Low (0-49) | *1.0* | **1.58**  **(1.18-2.12)** | **2.93**  **(2.23-3.83)** | **4.71**  **(3.54-6.25)** | **6.50**  **(4.87-8.67)** |
| Moderate (50-79) | *1.0* | 1.06  (0.89-1.26) | **1.46**  **(1.25-1.70)** | **1.64**  **(1.36-1.97)** | **2.23**  **(1.83-2.71)** |
| High (80-100) | *1.0* | *1.0* | *1.0* | *1.0* | *1.0* |
| ^a^social risks defined as: low family income (PIR<1/PIR >1), low education level (<HS/>HS), minority race/ethnic group (non-white status/white status), single-living status (unmarried/married), uninsured (uninsured/insured), unemployed (unemployed/employed)  ^b^Life’s Essential 8 Scores calculated from blood pressure, cholesterol, glucose, body mass index, smoking, physical activity, diet, and sleep)  ^c^adjusted for age and gender  95% CI: 95% Confidence Interval; Bold, Statistical significance: *P<0.05* | | | | | |

| **Table S4. Odds ratios for Life's Essential 8 components associated with individual**  **social risk factors among all participants (N= 21,625))** | | | | | | |
| --- | --- | --- | --- | --- | --- | --- |
| **Characteristics** | low family income | low education level | minority race/ethnic group | single-living status | uninsured | unemployed |
| Blood sugar | | | | | | |
| Low (0-49) | **1.25**  **(1.05–1.50)** | **1.53**  **(1.27–1.84)** | **2.30**  **(2.04–2.59)** | 1.08  (0.95–1.23) | **0.75**  **(0.63–0.89)** | **1.34**  **(1.15–1.56)** |
| Moderate (50-79) | **1.16**  **(1.01–1.33)** | **1.27**  **(1.11–1.46)** | **1.64**  **(1.50–1.80)** | 0.94  (0.85–1.04) | 0.90  (0.80–1.03) | 0.95  (0.86–1.05) |
| High (80-100) | *1.0* | *1.0* | *1.0* | *1.0* | *1.0* | *1.0* |
| Cholesterol | | | | | | |
| Low (0-49) | 1.06  (0.92–1.22) | 1.07  (0.94–1.21) | 1.06  (0.98–1.16) | **0.83**  **(0.76–0.91)** | 1.12  (0.98–1.28) | **0.87**  **(0.80–0.95)** |
| Moderate (50-79) | 0.98  (0.86–1.13) | 1.09  (0.95–1.25) | 0.98  (0.90–1.07) | **0.73**  **(0.66–0.81)** | **1.17**  **(1.03–1.32)** | **0.75**  **(0.67–0.84)** |
| High (80-100) | *1.0* | *1.0* | *1.0* | *1.0* | *1.0* | *1.0* |
| Blood pressure | | | | | | |
| Low (0-49) | 1.18  (0.99–1.42) | **1.29**  **(1.12–1.48)** | **1.54**  **(1.34–1.78)** | **1.16**  **(1.05–1.28)** | 1.02  (0.87–1.21) | 0.98  (0.87–1.10) |
| Moderate (50-79) | 1.03  (0.88–1.20) | 1.14  (0.98–1.32) | 1.07  (0.98–1.18) | **1.11**  **(1.01–1.21)** | 1.10  (0.96–1.26) | **0.82**  **(0.75–0.90)** |
| High (80-100) | *1.0* | *1.0* | *1.0* | *1.0* | *1.0* | *1.0* |
| Smoking | | | | | | |
| Low (0-49) | **1.83**  **(1.63–2.04)** | **1.67**  **(1.40–1.97)** | **0.54**  **(0.48–0.60)** | **1.63**  **(1.46–1.81)** | **1.96**  **(1.70–2.25)** | **1.30**  **(1.14–1.48)** |
| Moderate (50-79) | 1.03  (0.91–1.17) | **1.20**  **(1.05–1.36)** | **0.59**  **(0.52–0.66)** | 0.98  (0.86–1.13) | 1.05  (0.87–1.26) | 0.92  (0.82–1.05) |
| High (80-100) | *1.0* | *1.0* | *1.0* | *1.0* | *1.0* | *1.0* |
| Physical activity | | | | | | |
| Low (0-49) | **1.16**  **(1.03-1.30)** | **1.58**  **(1.39-1.79)** | **1.33**  **(1.23-1.44)** | 1.09  (1.00-1.20) | 1.10  (0.96-1.25) | **1.22**  **(1.12-1.34**) |
| Moderate (50-79) | 1.01  (0.85-1.19) | 1.07  (0.93-1.23**)** | **1.17**  **(1.05-1.29)** | **0.74**  **(0.67-0.82)** | 0.93  (0.71-1.22) | 1.09  (0.89-1.32) |
| High (80-100) | *1.0* | *1.0* | *1.0* | *1.0* | *1.0* | *1.0* |
| Diet | | | | | | |
| Low (0-49) | **1.23**  **(1.09–1.38)** | **0.87**  **(0.76–0.99)** | **0.82**  **(0.74–0.92)** | 1.10  (1.00–1.20) | 1.07  (0.93–1.23) | 1.13  (1.00–1.28) |
| Moderate (50-79) | 1.00  (0.86–1.16) | **0.87**  **(0.76–0.99)** | **0.81**  **(0.72–0.91)** | 0.97  (0.85–1.10) | 1.15  (0.96–1.39) | 1.01  (0.86–1.18) |
| High (80-100) | *1.0* | *1.0* | *1.0* | *1.0* | *1.0* | *1.0* |
| Sleep | | | | | | |
| Low (0-49) | **1.50**  **(1.27–1.77)** | 1.05  (0.88–1.25) | **1.79**  **(1.54–2.07)** | **1.51**  **(1.31–1.73)** | 1.17  (0.97–1.40) | 0.98  (0.84–1.15) |
| Moderate (50-79) | **1.36**  **(1.22–1.51)** | **1.33**  **(1.17–1.52)** | **1.31**  **(1.20–1.42)** | **1.35**  **(1.25–1.45)** | 1.01  (0.89–1.15) | 1.13  (1.00–1.27) |
| High (80-100) | *1.0* | *1.0* | *1.0* | *1.0* | *1.0* | *1.0* |
| High body mass index | | | | | | |
| Low (0-49) | 1.13  (0.98–1.32) | 1.07  (0.94–1.22) | **1.24**  **(1.10–1.41)** | **0.84**  **(0.75–0.94)** | 1.00  (0.84–1.19) | **0.86**  **(0.77–0.95)** |
| Moderate (50-79) | 1.01  (0.85–1.19) | 1.07  (0.93–1.23) | **1.17**  **(1.05–1.29)** | **0.74**  **(0.67–0.82)** | 1.08  (0.92–1.26) | **0.87**  **(0.79–0.96**) |
| High (80-100) | *1.0* | *1.0* | *1.0* | *1.0* | *1.0* | *1.0* |
